# 

**Published:** 1915-03

**Authors:** 


### THE BRISTOL
### Medico-Chirurgical Journal

A JOURNAL OF THE MEDICAL SCIENCES FOR THE WEST OF ENGLAND AND SOUTH WALES

*PUBLISHED UNDER THE AUSPICES OF*

*THE BRISTOL MEDICO-CHIRURGICAL SOCIETY.*

1133 / 1310
524385

EDITOR:

P. WATSON-WILLIAMS, M.D.

WITH WHOM ARE ASSOCIATED
J. MICHELL CLARKE, M.A., M.D., LL.D., J. LACY FIRTH, M.S., M.D.
AND JAMES SWAIN, M.S., M.D.

ASSISTANT-EDITOR:
J. A. NIXON, B.A., M.D.

EDITORIAL SECRETARY:
J. M. FORTESCUE-BRICKDALE, M.A., M.D.

"Scire est nescire, nisi id me

Scire alius sciret."

*VOL. XXXIII.*

BRISTOL: J. W. ARROWSMITH LTD.
LONDON: J. & A. CHURCHILL, 7 GREAT MARLBOROUGH STREET.
1915.

R31
B8

J.W. Arrowsmith Ltd. Publishers Bristol

### CONTENTS OF VOLUME XXXIII.

Page

Intestinal Stasis. By Sir WM. ARBUTHNOT LANE, Bart., M.S., F.R.C.S. .. .. .. .. .. .. .. .. 1

On Toxic Polyneuritis of the Motor Type. By J. MICHELL CLARKE, M.A., M.D., LL.D., F.R.C.P. .. .. .. .. .. 9

On the Pernasal Operation for Frontal Sinus Suppuration. By P. WATSON-WILLIAMS, M.D. Lond. .. .. .. .. 24

Drainage Continu par le Gaz Oxygène. Par M. LE DOCTEUR RENOIRDRE .. .. .. .. .. .. .. .. 39

An Outbreak of Institutional Dysentery due to the Y. Bacillus. By R. H. NORGATE, M.R.C.S., and I. WALKER HALL, M.D. .. 44

Cotton-Seed Dermatitis and its Cause, Pediculoides Ventricosus. By J. A. NIXON, M.B. Cantab., F.R.C.P. .. .. .. 73

Neuritis: its Definition and Successful Treatment. By NAUNTON DAVIES, F.R.C.S. .. .. .. .. .. .. .. 81

Women Doctors with the Army in the Field. By HELEN B. HANSON, M.D., B.S. Lond. .. .. .. .. .. .. .. 88

The Clinical Examination of Stools. By G. HELY-HUTCHINSON ALMOND, M.A., B.M., B.Ch. (Oxon.), M.R.C.S. (Eng.), L.R.C.P. Lond. .. .. .. .. .. .. .. .. .. 95

Presidential Address .. .. .. .. .. .. .. 129

Cardiac Diseases and Disorders in Warfare. By CAREY COOMBS, M.D., M.R.C.P. Lond. .. .. .. .. .. .. 149

Removal of Bullets and other Metallic Foreign Bodies. By R. G. POOLE LANSDOWN, M.D., B.S. Durham .. .. .. .. 157

The Long Fox Lecture: The Evolution of the Sense of Sight. By F. RICHARDSON CROSS, M.B. Lond. .. .. .. .. 163

Gunshot Injuries to the Eyes. By E. H. E. STACK, M.B., F.R.C.S. .. 198

5.m. 26/viii/20

CONTENTS.

| | Page |
|---|---|
| Progress of the Medical Sciences .. .. .. | 54, 108, 206 |
| Reviews of Books .. .. .. .. .. | 63, 114, 209 |
| Editorial Notes .. .. .. .. .. .. .. | 65, 218 |
| Therapeutic Notes .. .. .. .. .. .. .. | 68 |
| Meetings of Societies .. .. .. .. .. .. | 71, 226 |
| Obituary .. .. .. .. .. .. .. .. | 123, 228 |
| Local Medical Notes .. .. .. .. .. | 72, 126, 232 |

J. Lacy Firth,

J. M. Fortescue-Brickdale,

*Acting Hon. Sec.*

---

### Local Medical Notes.

**University of Bristol.**—Professor A. F. Stanley Kent, Professor of Physiology, has been awarded the Honorary Degree of D.Sc. by the University of Oxford.

EXAMINATION RESULTS.

Students of the University have recently passed the undermentioned examinations: —

University of Bristol.—*M.B., Ch.B., Intermediate Examination:* Evelyn B. Salter, J. D'A. Champneys. *D.P.H. Examination:* W. A. Reynolds.

Conjoint Examining Board of England.—*Medicine:* W. K. Richards, C. N. Vaisey.* *Surgery:* W. K. Richards.* *Midwifery:* J. W. Gilbert,* G. S. Terry.*

L.D.S., R.C.S. Eng.—*Final Examination:* W. A. Culverwell,* W. J. Milton,* Marjorie J. White.*

APPOINTMENT.

A. G. T. Fisher, M.R.C.S., L.R.C.P., has been appointed Senior Resident Officer at the Bristol General Hospital.

* Qualified.

### Bristol Medico-Chirurgical Society.

### President.

W. H. C. NEWNHAM, M.A., M.B. Cantab.

### President-Elect.

G. PARKER, M.A., M.D. Cantab.

### Committee.

*Ex-Officio*
- P. WATSON-WILLIAMS, M.D. Lond. (Editor of *Journal* and Retiring President).
- C. K. RUDGE, M.R.C.S. (Hon. Librarian).
- J. M. FORTESCUE-BRICKDALE, M.A., M.D. Oxon. (Editorial Secretary).

*Elected.*

R. SHINGLETON SMITH, M.D. Lond. } Ex-Presidents.
G. MUNRO SMITH, M.D., Bristol. } Ex-Presidents.
I. WALKER HALL, M.D. Vict.
J. LACY FIRTH, M.D., M.S. Lond.
J. O. SYMES, M.D. Lond.
L. E. V. EVERY-CLAYTON, M.D., B.S. Lond.
H. S. BALLANCE, M.D. Lond.
F. H. EDGEWORTH, B.A., M.D. Cantab., D.Sc. Lond.

### Honorary Secretary.

A. J. WRIGHT, M.B., B.S. Lond.

Medical Practitioners wishing to join the Society are requested to communicate with the Acting Hon. Secretary, Dr. FORTESCUE-BRICKDALE, 52 Pembroke Road, Clifton. The Annual Subscription is 21/-.

---

*Extracts from Journal By-laws.*

4.—The *Journal* shall be delivered free to Subscribers and also to Members of the Society whose subscriptions are not in arrear.

6.—The Annual Subscription to the *Journal* for those who pay before the 1st of July shall be 5/-, post free, or 6/- if paid after that date.

---

Communications intended for insertion in the *Journal* should be sent to the Editor, Dr. WATSON-WILLIAMS, 2 Rodney Place, Clifton, Bristol.

Books for Review and Exchange Journals should be sent to the Assistant-Editor, *Bristol Medico-Chirurgical Journal*, Medical Library, The University, Bristol.

Communications referring to the delivery of the *Journal* should be sent to the Editorial Secretary, Dr. FORTESCUE-BRICKDALE, 52 Pembroke Road, Clifton, Bristol, to whom Subscriptions are to be paid *in advance*.

Cases for Binding Vols. I—XXXII are now ready, and may be obtained, price 7*d*. each (postage 2*d*. extra), upon application to J. W. ARROWSMITH LTD., Quay Street, Bristol; or the *Journal* will be bound, including Case, for 1/3 per volume.

### Bristol Medico-Chirurgical Society.

### PAST PRESIDENTS.

1874—75. FREDERICK BRITTAN, M.D., B.A. Dub.
1875—76. ROBERT WILLIAM COE.
1876—77. SAMUEL MARTYN, M.D. Ed.
1877—78. AUGUSTIN PRICHARD, M.D. Berlin.
1878—79. HENRY EDWARD FRIPP, M.D. Würzburg.
1879—80. WILLIAM MICHELL CLARKE.
1880—81. JOSEPH GRIFFITHS SWAYNE, M.D. Lond.
1881—82. EDWARD LONG FOX, M.D. Oxon.
1882—83. JAMES GEORGE DAVEY, M.D. St. And.
1883—84. WILLIAM JOHNSTONE FYFFE, M.D., B.A. Dub.
1884—85. GEORGE FORSTER BURDER, M.D. Aberd.
1885—86. WILLIAM HENRY SPENCER, M.D., M.A. Cantab.
1886—87. FRANCIS POOLE LANSDOWN.
1887—88. LEMUEL MATTHEWS GRIFFITHS.
1888—89. ROBERT SHINGLETON SMITH, M.D., B.Sc. Lond.
1889—90. NELSON CONGREVE DOBSON, Ch.M. Bristol.
1890—91. SAMUEL HENRY SWAYNE.
1891—92. FRANCIS RICHARDSON CROSS, M.B.Lond., LL.D.Bristol.
1892—93. EDWARD MARKHAM SKERRITT, M.D., B.S., B.A. Lond.
1893—94. JAMES GREIG SMITH, M.B., C.M., M.A. Aberd., F.R.S.E.
1894—95. ALFRED JAMES HARRISON, M.B. Lond.
1895—96. ARTHUR WILLIAM PRICHARD.
1896—97. ALFRED EDWARD AUST LAWRENCE, M.D., C.M. Aberd.
1897—98. JOHN EDWARD SHAW, M.B., C.M. Ed.
1898—99. ROBERT ROXBURGH, M.B. Ed.
1899—00. WILLIAM HENRY HARSANT.
1900—01. DAVID SAMUEL DAVIES, M.D. Lond.
1901—02. BARCLAY JOSIAH BARON, M.B., C.M. Ed.
1902—03. GEORGE MUNRO SMITH, M.D. Bristol.
1903—04. JAMES PAUL BUSH, C.M.G., Ch.M. Bristol.
1904—05. REGINALD EAGER, M.D. Lond.
1905—06. JOHN DACRE.
1906—07. JAMES TAYLOR.
1907—08. HENRY WALDO, M.D., C.M. Aberd.
1908—09. JOHN MICHELL CLARKE, M.D. Cantab.
1909—10. JAMES SWAIN, M.D., M.S. Lond.
1910—11. BERTRAM MITFORD HERON ROGERS, B.A., M.D., B.Ch. Oxon.
1911—12. CHARLES A. MORTON.
1912—13. WALTER C. SWAYNE, M.D., B.S. Lond. and Bristol.
1913—14. P. WATSON-WILLIAMS, M.D. Lond.

1915.

### LIST OF SUBSCRIBERS

TO THE

### Bristol Medico-Chirurgical Journal.

### HONORARY MEMBERS OF THE SOCIETY.

| | |
|---|---|
| Owen, Sir Isambard, D.C.L., M.D. | Hereford House, Clifton. |
| Dobson, N. C., Ch.M., F.R.C.S. ... | 16 College Road, Clifton. |
| Griffiths, L. M., M.R C.S. ... ... | 11 Pembroke Road, Clifton. |
| Smith, R. Shingleton, M.D., B.Sc., F.R.C.P. ... ... ... ... ... ... | Deepholm, Clifton Park, Clifton. |

### SUBSCRIBERS.

*Those marked * are Members of the Society.*

| | |
|---|---|
| Ackland, J. McKno ... ... ... ... ... ... | 1 Barnfield Crescent, Exeter. |
| *Ackland, W. R. ... ... ... ... ... ... ... ... | 5 Rodney Place, Clifton. |
| Adye, W. J. A. ... ... ... ... ... ... ... ... ... | Church House, Bradford, Wilts. |
| Aldous, George F. ... ... ... ... ... ... ... | Charlton House, Mannamead, Plymouth. |
| *Alexander, D.A., M.B., Ch.B. Vict.... ... ... | 30 Berkeley Square, Clifton. |
| Alford, H. J., M.D. Lond.... ... ... ... ... ... | 19 Park Street, Taunton. |
| *Almond, G. H. H., M.A., M.B., B.Ch. Oxon. | 6 Brock St., Bath. |
| *Askins, R.A., M.D., B.Ch., B.A.O. Dubl. ... | Arlington Villas, Clifton. |
| *Aubrey, T., M.B. Lond. ... ... ... ... ... | Bitton, near Bristol. |
| Aveline, H. T. S. ... ... ... ... ... ... ... ... | Somerset Asylum, Taunton. |

| | |
|---|---|
| *Baker, Lily A., M.B., B.Ch., B.A.O., R.U.I. ... | 3 Whiteladies Road, Clifton. |
| *Ballance, H. Stanley M.D. Lond. ... ... | 4 Royal Terrace, Weston-super-Mare. |
| Barber, M. C. ... ... ... ... ... ... ... ... | Staple Hill, Bristol. |
| Baretti, T. G. L'Enardi ... ... ... ... ... ... | 49 Royal York Crescent, Clifton. |
| *Barker, G. H., M.D., B.Sc. Lond. ... ... ... | 124 Redland Road, Bristol |
| *Baron, Barclay J., M.B., C.M. Ed.... ... ... | 16 Whiteladies Road, Clifton. |
| Basset, Walter ... ... ... ... ... ... ... ... | 24 Stow Hill, Newport, Mon. |

### LIST OF SUBSCRIBERS.

| | |
|---|---|
| Beaver, R. A., M.D. | Wotton-under-Edge. |
| *Benson, J. R. | 11 The Circus, Bath. |
| *Bergin, F. G. | 31 Oakfield Road, Clifton. |
| Bernard, C. | 2 Spencer Terrace, Fishponds, Bristol. |
| Berry, F. C., M.D., B.Ch., B.A. Dub. | Burnham, Somerset. |
| Berry, James, M.B., B.S. Lond. | 21 Wimpole Street, London, W. |
| Biamonti, Sebastiano | 27 Via Balbi, Genoa, Italy. |
| *Blacker, A. E. | 20 Victoria Square, Clifton. |
| Blackett, J. F., M.D., B.S. Lond. | Audley, Newcastle, Staffs. |
| *Blagg, Arthur F., M.D. Durh. | 28 Caledonia Place, Clifton. |
| Bodman, Hervey, M.D. Lond. | 9 Whiteladies Road, Clifton. |
| *Bowker, G. E., M.D. Edin. | 11 North Parade, Bath. |
| Boyce, James G. | The Old Hall, Biddenden, Kent. |
| *Brasher, C. W. J., M.B., Ch.B. Bristol | 17 Oakfield Road, Clifton. |
| *Brew, R. H. | Chew Magna. |
| *Bristowe, H. C., M.D. Lond. | Wrington, Somerset. |
| *Brown, William, M.D., C.M. Glasg. | Park View, Fishponds, Bristol. |
| *Bullen, F. St. John | 3 Richmond Park Road, Clifton. |
| *Burdon-Cooper, J., M.D., B.Sc. Durh. | 12 The Circus, Bath. |
| Burroughs, E. F. H. | 27 Burlington Road, Redland, Bristol. |
| *Bush, J. Paul, C.M.G., Ch.M. Bristol | Vyvyan House, Clifton Park, Clifton. |

| | |
|---|---|
| *Cadman, E. S. R., M.D., C.M. Edin. | 22 Trelawney Road, Cotham, Bristol. |
| *Cairns, F. J. | Devon House, Whitehall Road, Bristol. |
| *Carling, A., M.A., M.B., B.C. Cantab. | 12 Great George Street, Bristol. |
| *Carter, T. M., M.D. Durh. | Burnley, Westbury-on-Trym. |
| *Carwardine, Thomas, M.B., M.S. Lond. | 16 Victoria Square, Clifton. |
| *Cave, Edward J., M.D. Lond. | 20 Circus, Bath. |
| *Charles, J. R., M.A., M.D., B.C. Cantab. | 12 Victoria Square, Clifton. |
| *Chitty, H., M.S. Lond. | 46 Pembroke Road, Clifton. |
| *Christofferson, P. E., M.B., Ch.B. Bristol | 14 Buckingham Place, Clifton. |
| Chute, Henry M. | King William's Town, S. Africa. |
| *Clarke, C., M.B., B.S. Lond. | Brompton Hospital, London. |
| *Clarke, John Michell, M.D., M.A. Cantab. | 28 Pembroke Road, Clifton. |
| *Clarke, R. C., M.B., Ch.B. Bristol | Amherst House, Clifton Park, Clifton. |
| Clowes, E. F. | Wotton-under-Edge. |
| Connellan, Lieut. P. S., I.M.S. | c/o Grindlay & Co., 54 Parliament Street, London. |
| *Cooke, H. W. | The Royal Infirmary, Bristol. |
| *Coombs, Carey, M.D. Lond. | 3 Pembroke Road, Clifton. |
| *Cooper, William Russell | 32 Whiteladies Road, Clifton. |
| *Corfield, C. | 12 Pembroke Road, Clifton. |
| Cossham, W. R., M.D., C.M. Aberd. | Cirencester. |
| Cotton, William, M.D., C.M., M.A. Ed. | 231 Gloucester Road, Bristol. |
| Coulter, R. J., M.B. Dub. | 11 Clytha Park Road, Newport, Mon. |
| Cridland, A. B. | 52 Waterloo Road, Wolverhampton. |
| *Cross, F. Richardson, M.B. Lond., LL.D. Bristol | Worcester House, Clifton. |
| *Crossman, F. W. | Hambrook, Gloucestershire. |
| *Crouch, C. Percival, M.B. Lond. | Villa Rosa, Weston-super-Mare. |

### LIST OF SUBSCRIBERS.

| | |
|---|---|
| *Dacre, John | 14 Eaton Crescent, Clifton. |
| *Davies, D. S., M.D. Lond., D.P.H. Cantab. | 6 Lansdown Place, Clifton. |
| Dening, Edwin | Manor House, Stow-on-the-Wold. |
| Devine, H., M.D., Lond. | The Asylum, Milton, Portsmouth. |
| *Dixon, T. B. | 134 Coronation Road, Bristol. |
| *Dobbyn, M. G. | 85A Redland Road, Bristol. |
| *Dowling, E. A. G. | 5 Rodney Place, Clifton. |
| *Drapes, T. L., M.B., B.C., B.A. Cantab. | St. Maur, Beaufort Square, Chepstow. |
| *Duckett, A. H., M.B. | 27 Bloomfield Avenue, Bath. |
| Dunbar, Eliza L. Walker, M.D. Zürich | 9 Oakfield Road, Clifton. |
| Dymoke, F. | 21 Cotham Road, Bristol. |

| | |
|---|---|
| *Eager, Reginald, M.D. Lond. | |
| *Edgeworth, F. H., M.D., B.C., B.A. Cantab., D.Sc. Lond. | 4 Richmond Park Road, Clifton. |
| *Edridge, Ray | Lyn Vale, St. Mark's Place, Bath. |
| Edwards, C. D., B.A., M.D. Cantab. | The Corderies, Chalford, Glos. |
| Elder, William | 4 Trelawney Road, Cotham, Bristol. |
| Elliot Bros. Ltd. | *Australian Pharmaceutical Notes & News*, O'Connell Street, Sydney, N.S.W. |
| *Elliott, Christopher, M.D., B.A. Dub. | 102 Pembroke Road, Clifton. |
| Elliott, F. Percy, M.B., C.M. Aberd. | 113 Grove Road, Walthamstow, London, N.E. |
| *Eskell, B.C., M.B., Ch.B. Bristol | 26 Zetland Road, Bristol. |
| *Every-Clayton, L. E. V., M.D. Lond | Rosslyn, Clevedon, Somerset. |

| | |
|---|---|
| *Faill, C. J. C. | Tuberculosis Dispensary, Redcliffe Parade, Bristol. |
| Farnfield, W. W. | South Lynn, Gillingham, Dorset. |
| *Fawn, G. F., B.D.S. Bristol | 25 Whiteladies Road, Clifton. |
| *Fells, A., M.B., C.M. Edin. | 107 Ashley Road, Bristol. |
| *Firth, J. L., M.D., M.S. Lond. | 8 Victoria Square, Clifton. |
| *Flemming, A. L., M.B., Ch.B. Bristol | 48 Pembroke Road, Clifton. |
| *Flemming C. E. S. | Manvers House, Bradford-on-Avon. |
| *Fortescue-Brickdale, J. M., M.A., M.D., B.Ch. Oxon. | 52 Pembroke Road, Clifton. |
| Foss, E. V., M.D., Ch.B. Bristol | Clouds Hill House, St. George, Bristol. |
| *Fraser, Forbes | 2 The Circus, Bath. |
| Freeman, J., M.D. Brux. | Waterloo Villa, Queen's Road, Clifton. |

| | |
|---|---|
| *Galpin, P. A., M.D., B.S. Durh. | 47 Greville Road, Southville. |
| Garland, E. B., M.B., C.M. Edin. | 114*a* Hampton Road, Redland, Bristol. |
| Gee, C. A. H., M.B., Ch.B. Bristol | Evercreech, Somerset. |
| Genge, T. T. | 21 Whiteladies Road, Bristol. |

| | |
|---|---|
| *Gerrish, D. S. ... ... ... ... ... ... ... ... | 322 Church Road, St. George, Bristol. |
| *Gibbs, A. N. Godby ... ... ... ... ... ... ... | 52 Whiteladies Road, Clifton. |
| *Goodden, H. W., M.B., Ch.B. Bristol ... ... | The Royal Infirmary, Bristol. |
| Gough, B. B. ... ... ... ... ... ... ... ... ... | Compton Martin, Nr. Bristol. |
| *Grant, J., M.B. ... ... ... ... ... ... ... ... | The Royal Infirmary, Bristol. |
| *Gratte, C. Brooke ... ... ... ... ... ... ... | 19 Gold Tops, Newport, Mon. |
| *Green, T. A., M.D., C.M. Edin. ... ... ... | 33*a* Whiteladies Road, Clifton. |
| Green-Armytage, Capt. V. B., I.M.S. ... ... | c/o T. Cook & Son, Calcutta. |
| *Greer, W. J. ... ... ... ... ... ... ... ... ... | 19 Gold Tops, Newport, Mon. |
| Griffiths, Cornelius A. ... ... ... ... ... | 6 Windsor Place, Cardiff. |
| *Griffiths, J. S. ... ... ... ... ... ... ... ... | 20 Redland Park, Bristol. |
| *Groves, E. W. H., M.D., M.S. Lond. ... ... | 16 Richmond Hill, Clifton. |

| | |
|---|---|
| *Hailes, Clements D. G., M.D., C.M. Ed. ... | 27 Alma Road, Clifton. |
| *Hall, E. G., M.B. Lond. ... ... ... ... ... | 42 Apsley Road, Clifton, Bristol. |
| *Hall, I. Walker, M.D., Ch.B.Vict. ... ... | Linden Gate, Clifton Down Road, Clifton. |
| Handfield-Jones, Montagu, M.D. Lond. ... | 35 Cavendish Square, London, W. |
| *Harris, H. Elwin, B.A., M.B. Cantab. ... ... | 13 Lansdown Place, Clifton. |
| *Harsant, W. H. ... ... ... ... ... ... ... ... | Tower House, Clifton Down Road, Clifton. |
| *Harty, J. P. I., M.B., B.Ch., B.A.O., R.U.I. | 5 Westbourne Place, Clifton. |
| Hawes, C. S. ... ... .. ... ... ... ... ... ... | 8 Elsworthy Road, London, N.W. |
| *Hawes, I. H., M.B., B.S. Durh. ... ... ... | Wick, Glos. |
| Hayman, Alfred G. ... ... ... ... ... ... | Hapsford House, near Frome. |
| *Hayman, C. A., M.D. St. And. ... ... ... ... | Kingston Villa, Richmond Hill, Clifton. |
| Hayman, Howard ... ... ... ... .. ... ... | 3 Clifton Park, Clifton. |
| Hele, T. S., M.A., M.B. ... ... ... ... ... | Emmanuel College, Cambridge. |
| Henson, W. J. ... ... ... ... ... ... ... ... | 2 Derby Street, London, W. |
| *Herapath, C. E. K., M.D. Lond. ... ... ... | 12 Brunswick Square, Bristol. |
| *Herapath, C. K. C. ... ... ... ... ... ... ... | Cotham Park, Bristol. |
| Hill, Hedley, M.D. Durh. ... ... ... ... | 1 Whiteladies Road, Clifton. |
| *Hill, R. A. P., M.D. ... ... ... ... ... ... ... | 14 Florence Park, Redland. |
| *Hill, Walter J. ... ... ... ... ... ... ... ... | The Brow, Clevedon, Som. |

Hospital, Bristol General (*Secretary*, T. W. Gregg).

| | |
|---|---|
| *Iles, A. E., M.B., B.S. Lond. ... ... ... ... | Eye Hospital, Bristol. |
| *Imlay, G. A., M.B., C.M. Aberd. ... ... ... | 1 Cotham Park, Bristol. |

Infirmary, Bristol Royal (*Secretary*, W. E. Budgett).

| | |
|---|---|
| Ingle, C. Durban ... ... ... ... ... ... ... | Somerton, Somerset. |

| | |
|---|---|
| *James, C. W. W. ... ... ... ... ... ... ... | 246 Wells Road, Knowle, Bristol. |
| Jamison, A., M.B., B.Ch., M.A. R.U.I. ... ... | Ardenvohr Woodstock Road, Belfast. |
| *Joll, C.A., M.B., M.S. Lond. ... ... ... ... | Royal Free Hospital, Gray's Inn Road, London, W.C. |

| | |
|---|---|
| *Jones, J. Ellington | 8 Chantry Road Clifton. |
| Joseph, A. Hill, M.D. Lond. | Bexhill-on-Sea. |

| | |
|---|---|
| *Kay-Mouat, J. R., M.A. Oxon., M.B., Ch.B. Bristol | 5 Gloucester Row, Clifton. |
| *Kemm, F. St. J. | 17 Ravenswood Road, Redland. |
| *Kerfoot, Stanley J. | 109 East Street, Bedminster, Bristol. |
| *Kerr, R. A., M.B., B.Ch. | Royal Infirmary, Bristol. |

| | |
|---|---|
| *Lace, F. | 5 Gay Street, Bath. |
| *Lansdown, R. G. P., M.B., B.S. Durh. | 39 Oakfield Road, Clifton. |
| *Lavers, Norman, M.D. Brux. | Bailbrook House, Bath. |
| *Lavington, C. C., M.B., B.S. Durh. | 1 Burlington Road, Redland, Bristol. |
| Lendon, A. A., M.D. Lond. | North Terrace, Adelaide, S. Australia. |
| *Lees, E. Leonard, M.D., C.M. Ed. | 1 Chesterfield Place, Clifton. |
| Leigh, W. W. | Treharris, Glamorganshire. |
| *Leonard, R. C., M.D. Ed. | 91 Coronation Road, Bristol. |
| *Lewis, D. J. | Winscombe, Som. |
| *Linton, Marion S., M.B. Lond. | 21 Oakfield Road, Clifton. |
| *Llewellyn, R. Ll. J., M.B. Lond. | 41 Gay Street, Bath. |
| *Longley, J. A. Noel, M.B., Ch.B. Birm. | 2 Portland Square, Bristol |
| *Lucas, J. J. S., M.D. Lond. | 186 Wells Road, Totterdown, Bristol. |

| | |
|---|---|
| MacDonald, P. W., M.D., C.M. Aberd. | Herrison, Dorchester. |
| Mackinnon, Charles, M.B., C.M., M.A. Glasg. | Davaar, Cirencester. |
| Maclean, Ewen J., M.D., C.M. Ed. | 12 Park Place, Cardiff. |
| Maclean, J. Campbell, M.B., C.M. Ed. | Swindon House, Swindon, Wiltshire. |
| *Macphail, J. M., M.B., Ch.B. Ed. | Eastern Dispensary, Bath. |
| *MacWatters, J. C. | Almondsbury. |
| Marsh, O. E. Bulwer | Parkdale, Newport, Mon. |
| Marshall, Arthur L., M.B., B.A. Cantab. | 206 Upper Clapton Road, London, N.E. |
| *Martin, E. F., M.B. Ed. | 7 Royal Terrace, Weston-super-Mare. |
| *Martyn, G. J. King, M.D., B.C., B.A. Cantab. | 12 Gay Street, Bath. |
| Mason, S. B. | 12 Park Terrace, Pontypool. |
| Miall, C. L | Elm Hayes, Paulton, near Bristol. |
| *Mole, Harold F. | 19 Mortimer Road, Clifton. |

| | |
|---|---|
| *Moore, C. A., M.B., M.S. Lond. ... ... ... ... | 56 St. Paul's Road, Clifton. |
| More-O'Ferrall L. ... ... ... ... ... ... | 52 Cumberland Road, Bristol. |
| Morris, L. N. ... ... ... ... ... ... ... ... | 263 Stapleton Road, Bristol. |
| *Morris, Mary E. H., M.D. Lond. ... ... ... | 19 Gay Street. Bath. |
| *Morton, C. A. ... ... ... ... ... ... ... ... | 14 Vyvyan Terrace, Clifton. |
| Munden, Charles ... ... ... ... ... ... ... | Ilminster. |
| *Munro, J. M. H., D.Sc. Lond. ... ... ... ... | 12 Grosvenor Place, Bath. |
| *Myles, G T. ... ... ... ... ... ... ... ... | 7 Apsley Road, Clifton. |

| | |
|---|---|
| *Neild, Newman, M.B., B.Ch. Vict. ... ... ... | 9 Richmond Hill, Clifton. |
| *Newnham, W. H. C., M.B., M.A. Cantab. ... | 3 Lansdown Place, Victoria Square, Clifton. |
| *Nichols, F. C., M.B., Ch.B. Bristol ... ... ... | 38 Whiteladies Road, Clifton. |
| *Nixon, J. A., B.A., M.B., B.C. Cantab. ... ... | 7 Lansdown Place, Clifton. |
| *Nixon, J. H., M.D., B.S. Lond. ... ... ... | Morley House, Chippenham |
| *Noel-Cox, H. L., M.D. Durham ... ... ... | 11 Clifton Hill, Clifton. |

| | |
|---|---|
| Oppenheimer, Son & Co. Ltd. ... ... ... | 179 Queen Victoria Street, E.C. |
| *Ormerod, H. Lawrence, M.B., B.Ch. R.U.I. | 2 Henleaze Road, Westbury Park, Bristol. |
| *Orr-Ewing, H. J., M.B., B.S. Lond. ... ... | Royal Infirmary, Bristol. |

| | |
|---|---|
| Page, G. Shepley ... ... ... ... ... ... ... ... | 78 Old Market Street, Bristol. |
| *Parker, George, M.D., M.A. Cantab. ... ... | 14 Pembroke Road, Clifton. |
| Parkinson, Capt. G. S., R.A.M.C. ... ... ... | 25 Cheyne Court, Chelsea, London. |
| *Parsons, H. F.... ... ... ... ... ... ... ... | The Heath, Stoke Bishop, Bristol. |
| Parsons, J. H., M.B., B.S., D.Sc. Lond.... ... | 54 Queen Anne Street, London, W. |
| Paterson, Donald Rose, M.D., C.M. Ed. ... | 15 St. Andrew's Crescent, Cardiff. |
| Peake, G. Arthur ... ... ... ... ... ... ... | Alma House, Cheltenham. |
| Phillips, C. M., M.D. Brux. ... ... ... ... | Ravenslea, Kensington Hill, Brislington. |
| *Pickering, C. F. ... ... ... ... ... ... ... ... | 13 Berkeley Square, Bristol. |
| *Pineo, E. G. D.... ... ... ... ... ... ... ... | Richmond House, Langford, nr. Bristol. |
| *Porteous, H. B., M.B., Ch.B. Ed. ... ... ... | Newbury House, Weston-super-Mare. |
| Powell, J. J., M.D. Lond. ... ... ... ... ... | 2 Gloucester Road, Bishopston, Bristol. |
| Powell, William, M.B. Lond. ... ... ... ... | Hill Garden, Torquay. |

| | |
|---|---|
| Price, D. T. M.B. Lond. ... ... ... ... ... | Castle Cary, Somerset. |
| *Prichard, Arthur W. ... ... ... ... ... ... | 6 Rodney Place, Clifton. |
| *Prowse, Arthur B., M.D. Lond. ... ... ... | 5 Lansdown Place, Clifton. |

| | |
|---|---|
| Randolph, Charles ... ... .. ... .. ... | St. Michael's, Milverton, Somerset. |
| *Rattray, J. M., M.D., C.M., M.A. Aberd. ... | Frome. |
| *Rayner, D. C. ... ... ... ... ... ... ... ... | 9 Lansdown Place, Clifton, Bristol. |
| Rendall, John ... ... ... ... ... ... ... | Forestside, Lymington. |
| *Richardson, T. A. ... ... ... ... ... ... ... | 10 Clifton Park Road, Clifton. |
| Ritchie, Thomas P. ... ... ... ... ... ... | 27 Oakfield Road, Clifton. |
| *Robertson, D., M.B., Ch.B. Edin. ... ... ... | 188 Wells Road, Knowle. |
| *Rogers, B. M. H., M.D., B.Ch., B.A. Oxon. ... | 1 Victoria Square, Clifton. |
| *Rolfe, R., B.A., M.B., B.C. Cantab. ... ... | Park House, Avonmouth. |
| *Rose, F. H. ... ... ... ... ... ... ... ... ... | 13 Westfield Park, Clifton. |
| *Rossiter, George F., M.B. Lond. ... ... ... | Cairo Lodge, Weston-super-Mare. |
| *Roxburgh, Robert, M.B. Edin. ... ... ... | 29 Knightstone Road, Weston-super-Mare. |
| *Rudge, C. King ... ... ... ... ... ... .. | 145 Whiteladies Road, Bristol. |
| *Rutherford, J. M., M.B., C.M. Edin. .. ... | Brislington House, Bristol. |

| | |
|---|---|
| Scott, Baty- ... .. ... ... ... ... ... | 28 Belluton Road, Knowle, Bristol. |
| Seward, W. J., M.B. Lond. ... ... ... ... | 15 Chandos Avenue, Oakleigh Park, London, N. |
| *Short, A. R., M.D., B.S., B.Sc. Lond. ... ... | Montrose House, Queen's Road, Clifton. |
| *Short, L. J., M.D., B.Sc. Lond. ... ... ... | Westfield, South Road, Weston-super-Mare. |
| Smith, Capt. F. Shingleton, I.M.S. ... ... | c/o Grindlay & Co., Bombay. |
| *Smith, G. Cockburn, M.D. Brux. ... ... ... | 14 South Road, Newton Abbot. |
| *Smith, G. Munro, M.D. Bristol ... ... ... | 18 Apsley Road, Clifton. |
| Smith, L. Shingleton, B.A., M.B. Cantab.... | Camden Road, Brecon. |
| Smith, Rev. Reginald, M.A. Cantab. ... ... | The Vicarage, Walpole St. Andrew, Norfolk. |
| Smith, W. Steele, M.B. Lond. ... ... ... | 1 Wilbraham Rd., Chorlton-cum-Hardy, Manchester |
| *Smith, W. Alexander, M.B., M.A. Oxon. ... | 70 Pembroke Road, Clifton. |
| Society, Cardiff Medical (*Hon. Lib.*, Queen Street, Cardiff). | |
| Society, Manchester Medical ... ... ... | Medical Society's Library, The Owens College, Manchester. |

| | |
|---|---|
| *Stack, E. H. E., B.A., M.B., B.C. Cantab. ... | Arvalee, Clifton Down Road, Clifton. |
| Staple, J. D. ... ... ... ... ... ... ... ... | 153 Cheltenham Road, Bristol. |
| *Statham, R. S. S., M.D. Bristol ... ... ... | 5 Westbourne Place, Clifton. |
| *Stock, W. S. V., M.B., B.S. Lond. ... ... ... | 1 Mortimer Road, Clifton. |
| Stoddart, F. Wallis ... ... ... ... ... ... | Southey House, College Green Bristol |
| *Swain, James, M.D., M.S. Lond. ... ... ... | 4 Victoria Square, Clifton. |
| *Swayne, W. Carless, M.D. Lond. ... ... ... | 2 Clifton Park, Clifton. |
| *Symes, J. O., M.D. Lond. ... ... ... ... ... | 71 Pembroke Road, Clifton. |
| Symonds, H. P. ... ... ... ... ... ... ... | 35 Banbury Road, Oxford. |

| | |
|---|---|
| Taylor, A. L. ... ... ... .. ... ... ... ... | The Royal Infirmary, Bristol. |
| Taylor, H. N., M.A. Cantab., M.D. Dublin ... | Ebbw Vale. |
| *Taylor, James ... ... ... ... ... ... ... ... | 36 Alma Road, Clifton |
| *Taylor, J. W., M.D. Bristol ... ... ... ... | 8 West Redcliff Parade, Bristol. |
| *Taylor, S. H. S., B.A. Cantab., M.B., Ch.B.Ed. | Wray Croft, Lacock, Chippenham. |
| *Temple, G. H., M.B., C.M. Ed. ... ... ... | Ailanthus, Weston-super-Mare. |
| Thin, James ... ... ... ... ... ... ... ... | 54 & 55 South Bridge, Edinburgh. |
| *Thomas, J. D., M.B. Cantab. ... ... ... ... | Northwoods, Winterbourne. |
| Thomas, J. Lynn, C.B. ... ... ... ... ... ... | 21 Windsor Place, Cardiff. |
| *Thomas, J. Tubb, D.P.H. .. ... ... ... ... | The Halve House, Trowbridge. |
| *Thomas, R. E. M.D., B.S. Lond. ... ... ... | 3 Royal York Crescent, Clifton. |
| Thomson, Eustace B., M.D., C.M. Glasg. ... | Albany Place, Plymouth. |
| *Thomson, F. G., M.A., M.D. Lond. ... ... ... | 20 Gay Street, Bath. |
| *Thurnam, W. R., M.D., B.S. Durham ... ... | Nordrach-on-Mendip. |
| Tosswill, Leonard R., D.P.H., Cantab. ... | Devon County Council Offices, 14 Bedford Circus, Exeter. |

| | |
|---|---|
| Vachell, Herbert R., M.D., C.M. Aberd. ... | 18 Newport Road, Cardiff. |
| *Vickery, W. H. ... ... ... ... ... ... ... | 1 Trewartha Park, Weston-super-Mare. |

| | |
|---|---|
| *Waldo, Henry, M.D., C.M. Aberd. ... ... ... | 19 Pembroke Road, Clifton. |
| *Walker, Cyril H., M.B., B.A. Cantab. ... ... | 8 Oakfield Road, Clifton. |
| *Wallace, John ... ... ... ... ... ... ... | Lauriston, 15 Knightstone Road, Weston-super-Mare. |
| Wallace, J. T., M.B., B.Ch., B.A. R.U.I. ... | 48 Coronation Road, Bedminster, Bristol. |
| *Wallace, James W. ... ... ... ... ... ... | 1 Greville Road, Southville Bristol. |
| *Walters, C. F. ... ... ... ... ... ... ... | 5 Mortimer Road, Clifton. |
| *Waterhouse, R., M.D. Lond. .. ... ... ... | 25 Circus, Bath. |
| *Watson-Williams, P., M.D. Lond.... ... ... | 2 Rodney Place, Clifton. |
| Webber, William W. ... ... ... ... ... ... | Crewkerne. |

| | |
|---|---|
| *Whicher, A. H. | 115 Queen's Road, Clifton. |
| White, J. W. | Orchard House, Nailsea. |
| *White, Percy W. | College Green, Bristol. |
| Wigmore, J., M.D. R. U. I. | 16 Queen Square, Bath. |
| Willcox, R. Lewis | Warminster. |
| Willett, George G. D. | Keynsham, Somerset. |
| *Williams, E. C., M.B., B.C., B.A. Cantab. | 159 Whiteladies Road, Clifton. |
| Williams, H. Egerton | The Mount, Newport, Mon. |
| Williams, S. R., M.B. Lond. | Buckfastleigh, Devon. |
| *Williams, W. Roger | Morven, Walton-by-Clevedon. |
| *Williamson, G. Scott | The General Hospital, Bristol. |
| Willis, W. M. | 7 Regent Street, Nottingham. |
| *Wills, W. K., M.B., B.C. Cantab. | 19 Whiteladies Road, Clifton. |
| *Windsor-Aubrey, H. W. | Coronation Road, Bristol. |
| *Wood, Duncan | The General Hospital, Bristol. |
| *Wood, W. V. | Langford, near Bristol. |
| Woollcombe, Walter L. | 16 The Crescent, Plymouth. |
| *Wright, A. J., M.B., B.S. Lond. | 14 Victoria Square, Clifton. |
| Young, J., M.D. Glas. | 14 Chantry Road, Clifton. |

### OXYGEN

TELEPHONE 4490.

### ANY HOUR, DAY OR NIGHT.

### NITROUS OXIDE.

**Customers' Cylinders Promptly refilled.**

### The Western Dental Mfg. Co. (1914) Ltd.

**Depôt for Compressed Gases.**

*Telegrams:* "Forceps, Bristol." *Telephone* 4490.

*73, 75 & 79 Queen's Road,* **BRISTOL.**

Bristol Medico-Chirurgical Journal,
MARCH, 1915.

### EXCHANGE-LIST.

### AFRICA.

#### CAPE COLONY.

*Twice a month.*

*South African Medical Record.* Cape Town: Townshend, Taylor & Snashall.

### AMERICA.

#### ARGENTINE REPUBLIC.

*Weekly.*

*La Semana Médica.* Buenos Ayres: Junin 845–863.

#### CANADA

*Monthly.*

*The Canada Lancet.* Toronto: The Ontario Publishing Co. Ltd.

*The Canadian Medical Association Journal.* Toronto: Morang & Co. Ltd.

*Western Canada Medical Journal.* Winnipeg: Commonwealth Block.

#### MEXICO.

*Twice a month.*

*Gaceta Médica.* Mexico: Apartado postal, 517.

#### PERU.

*Twice a month.*

*La Crónica Médica.* Lima: Apartado postal, No. 629.

#### UNITED STATES.

*Weekly.*

*Boston Medical and Surgical Journal.* Boston: 101 Tremont Street.

*Medical Record.* New York: William Wood & Co.

*The Lancet-Clinic.* Cincinnati: University Building.

*Monthly.*

*American Journal of Surgery.* New York: 92 William Street.

*Archives of Pediatrics.* New York. E. B. Treat & Co.

*Buffalo Medical Journal.* Buffalo: 228 Summer Street.

*Bulletin of the Johns Hopkins Hospital.* Baltimore: The Johns Hopkins Press.

*California State Journal of Medicine.* San Francisco: Butler Building.

*Charlotte Medical Journal.* Charlotte: E. C. Register.

*New York State Journal of Medicine.* New York: 17 West 43rd Street.

*The American Journal of Obstetrics.* New York: William Wood & Co.

*The American Journal of Ophthalmology.* St. Louis: 316 Metropolitan Building.

*The Monthly Cyclopædia of Practical Medicine.* Philadelphia: The F. A. Davis Co.

*The Post-Graduate.* New York: 2nd Avenue and 20th Street.

*The Therapeutic Gazette.* Detroit: E. G. Swift.

*Quarterly.*

*The Alienist and Neurologist.* St. Louis: 3858 West Pine Boulevard.

*Irregularly.*

*The Journal of Experimental Medicine.* New York: The Rockefeller Institute for Medical Research.

*The Journal of Medical Research.* Boston: 240 Longwood Avenue.

*Yearly.*

*Smithsonian Report.* Washington: Government Printing Office.

*Studies from the Department of Pathology of the College of Physicians and Surgeons—Columbia University.* New York: 437 West 59th Street.

*Transactions of the American Association of Obstetricians and Gynecologists.* Philadelphia: William J. Dornan.

*Transactions of the American Dermatological Association.* New York: The Grafton Press.

*Transactions of the American Laryngological Association.* New York: The McConnell Printing Co.

*Transactions of the American Ophthalmological Society.* Hartford: The Case, Lockwood & Brainard Co.

*Transactions of the American Pediatric Society.* New York: E. B. Treat & Co.

*Transactions of the American Surgical Association.* Philadelphia: William J. Dornan.

*Transactions of the Association of American Physicians.* Philadelphia: William J. Dornan.

*Transactions of the College of Physicians of Philadelphia.* Philadelphia: William J. Dornan.

### ASIA

#### CHINA.

*Quarterly*

*The China Medical Missionary Journal.* Shanghai: 18 Peking Road.

#### INDIA.

*Monthly.*

*The Antiseptic.* Madras: U. Rama Rau.

*The Indian Medical Gazette.* Calcutta: Thacker, Spink & Co.

*Practical Medicine* Delhi: Egerton Street.

#### JAPAN.

*Monthly.*

*The Sei-I-Kwai Medical Journal.* Tokyo: Charity Hospital Medical College.

#### PHILIPPINE ISLANDS.

*Bi-monthly.*

*The Philippine Journal of Science* Manila: Bureau of Printing.

### AUSTRALASIA.

### NEW ZEALAND.

*Quarterly.*

[illegible] *Zealand Medical Journal.* Wellington: "The [illegible]minion" Office.

### EUROPE.

### AUSTRIA.

*Weekly.*

[illegible] *klinische Wochenschrift.* Vienna: Wilhelm [illegible]umüller.

### BELGIUM.

*Monthly.*

[illegible] *de l'Académie royale de Médecine de Belgique* [illegible]ssels: J. Goemaere.

### BRITISH ISLES.

*Weekly.*

[illegible] *Medical Journal.* London: 429 Strand.

[illegible] *Press and Circular.* London: A. A. Tindall.

[illegible] *Clinical Journal.* London: The Medical Pub-[illegible] Company, Limited.

[illegible] *Hospital.* London: The Scientific Press [illegible]mited).

[illegible] *Lancet.* London: 423 Strand.

[illegible] *Sanitary Record.* London: The Sanitary Pub-[illegible] Co., Ltd.

*Fortnightly.*

[illegible] *Journal of Tropical Medicine.* London: John [illegible] Sons & Danielsson, Ltd.

*Monthly.*

[illegible] *Edinburgh Medical Journal.* Edinburgh: Wm. Green [illegible] Sons.

[illegible] *Health.* London: The Society of Medical [illegible] of Health.

[illegible] *Birmingham Medical Review.* Birmingham: [illegible] Jones, Ltd.

[illegible] *British Journal of Dermatology.* London: [illegible] Lewis.

[illegible] *Glasgow Medical Journal.* Glasgow: Alex. [illegible]dougall.

[illegible] *Journal of Laryngology, Rhinology, and Otology.* [illegible]: Adlard & Son.

[illegible] *Medical Review.* London: 66 Finsbury Pave-[illegible]

[illegible] *Medical Chronicle.* Manchester: Sherratt & [illegible]es.

[illegible] *Polyclinic.* London: John Bale, Sons & Daniels-[illegible] Ltd.

[illegible] *Practitioner.* London: Howard Street, Strand.

[illegible] *Prescriber.* Edinburgh: 137 George Street.

[illegible] *Proceedings of the Royal Society of Medicine.* [illegible]: Longmans, Green & Co

*Quarterly.*

*Journal of Mental Science.* London: J. & A. Churchill.

*The British Journal of Tuberculosis.* London: Baillière, Tindall & Cox.

*The Caledonian Medical Journal.* Glasgow: Alex. Macdougall.

*The Northumberland and Durham Medical Journal.* Newcastle-upon-Tyne: T. M. Grierson.

*The West London Medical Journal.* London: Adlard & Son.

*Half-yearly.*

*The Liverpool Medico-Chirurgical Journal.* Liverpool: Medical Institution.

*Yearly.*

*Guy's Hospital Reports.* London: J. & A. Churchill.

*King's College Hospital Reports.* London: Adlard & Son.

*Ophthalmological Transactions.* London: J. & A. Churchill.

*St. Bartholomew's Hospital Reports.* London: Smith, Elder & Co.

*St. Thomas's Hospital Reports.* London: J. & A. Churchill.

*The Medical Annual.* Bristol: John Wright & Sons Ltd.

*The Medical Society's Transactions.* London: Harrison & Sons.

*The Transactions of the Edinburgh Obstetrical Society.* Edinburgh: Oliver and Boyd.

*The Westminster Hospital Reports.* London: Henry J. Glaisher.

*Transactions of the Edinburgh Medico-Chirurgical Society.* Edinburgh: James Thin.

*Transactions of the Royal Academy of Medicine in Ireland.* Dublin: John Falconer.

### DENMARK.

*Weekly.*

*Hospitalstidende.* Copenhagen: Jacob Lund.

### FRANCE.

*Three times a week.*

*Gazette des Hôpitaux.* Paris: 49 Rue St.-André des-Arts.

*Weekly.*

*Bulletin de l'Académie de Médecine.* Paris: Masson et Cie

*Gazette des Hôpitaux de Toulouse.* Toulouse: 13 Rue Ste-Ursule.

FRANCE—*Weekly* (continued).

*Gazette hebdomadaire des Sciences médicales de Bordeaux.* Bordeaux: 8 Rue Paul-Bert.

*L'Echo médical du Nord.* Lille: 128 Boulevard de la Liberté.

*Le Progrès médical.* Paris: 41 Rue des Ecoles.

*Revue hebdomadaire de Laryngologie, d'Otologie et de Rhinologie.* Bordeaux: G. Gounouilhou.

*Fortnightly.*

*Gazette des Praticiens.* Lille: Rue des Guinguettes, 18.

*Twice a month.*

*Gazette de Gynécologie.* Paris: 69 Rue d'Amsterdam.

*Revue de Thérapeutique médico-chirurgicale.* Paris: Félix Alcan.

*Monthly.*

*Journal d'Hygiène.* Paris: 79 Avenue de Wagram.

*Revue générale d'Ophtalmologie.* Paris: Masson et Cie.

GERMANY.

*Weekly*

*Zentralblatt für Chirurgie.* Leipzig: J. A. Barth.

*Zentralblatt für Gynäkologie.* Leipzig: J. A. Barth.

*Zentralblatt für innere Medicin.* Leipzig: J. A. Barth.

*Münchener medicinische Wochenschrift.* Munich: J. F. Lehmann.

GREECE.

*Monthly.*

*Ιατρική πρόοδος.* Syra: Renieri Brindesi.

*La Grèce médicale.* Syra: Renieri Brindesi.

HOLLAND.

*Weekly.*

*Nederlandsch Tijdschrift voor Geneeskunde.* Amsterdam: F. van Rossen.

ITALY.

*Twice a month.*

*Giornale Internazionale delle Scienze Mediche.* Naples[illegible] Piazza Plebiscito, 10.

*Bi-monthly.*

*Archivio di Ortopedia.* Milan: Via San Calimero, 3[illegible]

NORWAY.

*Monthly.*

*Norsk Magazin for Lægevidenskaben.* Christiania[illegible] Steen'ske Bogtrykkeri.

RUSSIA.

*Weekly.*

*Medycyna.* Warsaw: Niecala, 7.

*Twice a month.*

*St. Petersburger medicinische Zeitschrift.* S[illegible] Petersburg: K. L. Ricker.

*Monthly.*

*Finska Läkaresällskapets Handlingar.* Helsingfors[illegible] Mercators Tryckeri.

SPAIN.

*Weekly*

*El Siglo Médico.* Madrid: Magdalena 36.

SWEDEN.

*Bi-monthly.*

*Nordiskt Medicinskt Arkiv.* Stockholm: P. A. N[illegible]stedt & Söner.

TURKEY.

*Monthly*

*Gazette médicale d'Orient.* Constantinople: "Leva[illegible] Herald."

---

Books for Review and Exchange Journals should be addressed: The *Bristol Medico-Chirurgical Journal,* Medical Library, University, Bristol.

